# Deep semantic segmentation for the quantification of grape foliar diseases in the vineyard

**DOI:** 10.3389/fpls.2022.978761

**Published:** 2022-09-09

**Authors:** Ertai Liu, Kaitlin M. Gold, David Combs, Lance Cadle-Davidson, Yu Jiang

**Affiliations:** ^1^Department of Biological and Environmental Engineering, Cornell University, Ithaca, NY, United States; ^2^Plant Pathology and Plant-Microbe Biology Section, School of Integrative Plant Science, Cornell AgriTech, Cornell University, Geneva, NY, United States; ^3^Grape Genetics Research Unit, United States Department of Agriculture-Agricultural Research Service, Geneva, NY, United States; ^4^Horticulture Section, School of Integrative Plant Science, Cornell AgriTech, Cornell University, Geneva, NY, United States

**Keywords:** computer vision, plant disease, downy mildew, powdery mildew, machine learning, proximal sensing, vineyard management

## Abstract

Plant disease evaluation is crucial to pathogen management and plant breeding. Human field scouting has been widely used to monitor disease progress and provide qualitative and quantitative evaluation, which is costly, laborious, subjective, and often imprecise. To improve disease evaluation accuracy, throughput, and objectiveness, an image-based approach with a deep learning-based analysis pipeline was developed to calculate infection severity of grape foliar diseases. The image-based approach used a ground imaging system for field data acquisition, consisting of a custom stereo camera with strobe light for consistent illumination and real time kinematic (RTK) GPS for accurate localization. The deep learning-based pipeline used the hierarchical multiscale attention semantic segmentation (HMASS) model for disease infection segmentation, color filtering for grapevine canopy segmentation, and depth and location information for effective region masking. The resultant infection, canopy, and effective region masks were used to calculate the severity rate of disease infections in an image sequence collected in a given unit (e.g., grapevine panel). Fungicide trials for grape downy mildew (DM) and powdery mildew (PM) were used as case studies to evaluate the developed approach and pipeline. Experimental results showed that the HMASS model achieved acceptable to good segmentation accuracy of DM (mIoU > 0.84) and PM (mIoU > 0.74) infections in testing images, demonstrating the model capability for symptomatic disease segmentation. With the consistent image quality and multimodal metadata provided by the imaging system, the color filter and overlapping region removal could accurately and reliably segment grapevine canopies and identify repeatedly imaged regions between consecutive image frames, leading to critical information for infection severity calculation. Image-derived severity rates were highly correlated (r > 0.95) with human-assessed values, and had comparable statistical power in differentiating fungicide treatment efficacy in both case studies. Therefore, the developed approach and pipeline can be used as an effective and efficient tool to quantify the severity of foliar disease infections, enabling objective, high-throughput disease evaluation for fungicide trial evaluation, genetic mapping, and breeding programs.

## 1. Introduction

Disease management is critical to vineyard production. Among all grape diseases, downy mildew (DM) and powdery mildew (PM) cause considerable crop damages and economic losses annually. Both DM (*Plasmopara viticola*) and PM (*Erysiphe necator*) can infect grape leaves, canes, and clusters at nearly all stages, impacting the vineyard for multiple seasons (Pearson and Goheen, 1988; Thind et al., 2004). If not treated properly, DM infection can result in severe economic losses due to grapevine damages and vineyard replanting. PM infection also severely degrades grape quality and subsequent crop values, especially for the grape wine industry where nearly all wine grape varieties (*Vitis vinifera*) have very limited resistance to both diseases (Pearson and Goheen, 1988; Boso et al., 2019). As a result, fungicides have been widely used for grape DM and PM control (Gisi and Sierotzki, 2008), and in some cases, the usage can be overwhelming for potential risk assuage. Such excessive applications have been linked to adverse influences on human health (Kang et al., 2021), environment (Zubrod et al., 2019), and grower financial status. For example, fungicide applications to control powdery mildew can represent upwards of 70% of a vineyard's annual management expense. Additionally, intensive fungicide uses could lead to the increase of pathogen fungicide resistance and then disease control difficulty. Therefore, precision disease management is urgently needed to secure the productivity, profitability, and sustainability of the wine grape industry.

Efforts have been made to provide effective vineyard disease management and control strategies. The most straightforward strategy is to optimize fungicide application schedule based on disease occurrence to be detected and/or predicted at asymptomatic to very early stages. As disease infection and development are highly dependent on environmental factors, many studies have investigated the use of meteorological data to establish disease prediction models to assist in fungicide spray scheduling (Orlandini et al., 1993; Vercesi et al., 2000; Rossi et al., 2008; Chen et al., 2020; Sanghavi et al., 2021). While these models have shown some success and stakeholder adoption, they have two primary limitations. These models are usually site-specific and require onsite calibration to achieve reliable prediction accuracy. Sometimes, stakeholders may have not necessary resources (e.g., labor and instrumentation) to conduct the calibration, and therefore cannot readily adopt the models. More importantly, meteorological data are likely obtained at a very coarse scale (e.g., regional level) that cannot provide spatial details for disease evaluation in individual vineyards and/or research projects. While there are ongoing efforts in using other sensing technologies (e.g., optical sensing), asymptomatic or early disease detection is still a challenging task because diseases usually initiate from areas (e.g., lower and inner canopy) invisible to sensors for detection (Singh et al., 2020).

Fungicide trial evaluation and breeding of disease-resistant cultivars are alternative solutions that can provide long-term benefits for precision disease management. Fungicide trial evaluation aims to continuously monitor and evaluate the efficacy of potential fungicide treatments for a given region and crop, so that optimal treatments can be recommended to maximize the efficiency of fungicide applications and minimize adverse impacts such as the increase of pathogen fungicide resistance (Warneke et al., 2020; Campbell et al., 2021). Breeding disease resistant cultivars can provide natural protection to crops, which would dramatically reduce the need of intensive fungicide applications for risk assuage (Poland and Nelson, 2011; Di Gaspero et al., 2012). Both solutions require accurate disease infection evaluation in the field (Poland and Nelson, 2011; Di Gaspero et al., 2012; Chen et al., 2020; Warneke et al., 2020). It should be noted that the trial evaluation and breeding programs accept symptomatic disease detection and quantification because they focus on the difference of disease infection severity caused by either fungicides or genotypes.

Since most grape diseases appear firstly in grapevine canopies, foliar disease identification is a logical target to effectively characterize grape diseases with visible symptoms, such as grape DM and PM. Prior to leaf necrosis, DM infections typically look as yellow to brown “oil-spot” regions on the upper leaf surface, often with white fluffy sporulation on the lower leaf surface. PM infections usually appear as white, powder-like spots. Currently, human field scouting is the primary way to evaluate grape DM and PM infection. To guide human scouting, the Horsfall-Barratt scale has been proposed and adopted for disease severity assessments (Horsfall and Barratt, 1945), and the European and Mediterranean Plant Protection Organization (EPPO) standard has been widely adapted for fungicide efficacy evaluation (Buonassisi et al., 2017). However, field disease scouting is not only subjective (e.g., leaf sampling and visual inspection) but also requires skilled plant pathologists or experienced workforce who are often in low availability with high hiring cost. This has become a bottleneck for fungicide trial evaluation, research projects, and breeding programs related to disease resistance.

To overcome these issues, with the recent advances in optical sensing (particularly imaging techniques), researchers have developed proximal and remote sensing tools for disease identification and quantification. An intuitive method is to identify diseases of sampled leaves using handheld sensing devices (e.g., fluorescent signals) rather than human, subjective evaluation (Ghozlen et al., 2010; Lejealle et al., 2012; Latouche et al., 2015). In this way, knowledge and experience requirements of disease inspection can be dramatically reduced, so that common workforce with proper instrument operation training are able to conduct field disease scouting. However, active leaf sampling is still needed and can considerably affect evaluation performance. Furthermore, these handheld devices must be operated by human operators in the field, having limited scanning throughput and are thus not capable of passive disease monitoring. An alternative method is to assess diseases of whole crop canopies using autonomous sensing systems to avoid the leaf sampling process and improve scanning throughput. Commonly used systems include ground robots, unmanned aircraft systems (UAVs), manned aircraft, and satellites. While the aerial systems ranging from UAVs to Earth observations are capable of accurately measuring disease infections at scale (Barbedo, 2019; de Castro et al., 2021; Gold, 2021), they are constrained by the measurement resolution and sensing angles that are crucial to grape disease sensing. For instance, grape DM and PM firstly occur at the lower canopy and become mostly visible from the side canopy. Therefore, ground systems (e.g., robots) are considered more suitable options. Nonetheless, identifying disease infections in collected images is paramount to achieve accurate and rapid disease evaluation in the vineyard (Singh et al., 2018).

Image-based plant disease analysis has been intensively studied. Based on the core techniques used, studies can be classified into three categories: conventional image processing (IP)-based methods (Singh et al., 2020), conventional machine learning (ML)-based methods (Singh et al., 2016), and deep learning (DL)-based methods (Singh et al., 2018; Jiang and Li, 2020). Conventional IP-based methods have focused on the use of color, spectral, and texture information and filters to differentiate disease infections from healthy leaves and canopies. These methods have achieved good performance with advanced imaging modalities such as multispectral, hyperspectral (Bendel et al., 2020; Nguyen et al., 2021), and fluorescent imaging (Latouche et al., 2015). These methods are usually computationally efficient and provide pixel-level infection masks for infection severity calculation, but they need to be used concurrently with costly sensors and have limited generalizability to unseen datasets, presenting challenges of the model deployment in real world applications. ML-based methods can leverage features extracted using IP methods and learn decision rules (rather than predefined ones) for image classification and segmentation (Jian and Wei, 2010; Kaur et al., 2018; Mahmud et al., 2020). This addresses the model generalizability issue to a certain extent, but image feature designing and extraction are largely manually crafted (namely feature engineering), which could be suboptimal for unseen datasets. Many recent studies have reported DL-based methods for improved accuracy and robustness of analyzing plant disease images without feature engineering (Singh et al., 2018; Jiang and Li, 2020; Benos et al., 2021). The DL-based methods learn features through training datasets and have achieved state-of-the-art performance in image classification, detection, and segmentation. Semantic segmentation is preferred, because resultant segmentation masks contain both location and quantity information of disease infections at the pixel level, enabling accurate localization for treatment application and quantification of infection severity.

Specifically for grape DM and PM, Oberti et al. (2014) conducted the first experiment to thoroughly study the optimal viewing angles of sensing grape PM on leaves, and concluded that acute angles (30 to 50 degrees) from the leaf surface provide ample information for grape PM detection. Abdelghafour et al. (2020) developed an imaging system with strobe light illumination for vineyard image acquisition, and a traditional probability based method for feature extraction and grouping to segment grape DM infections in collected images. While the method was reliable on the reported dataset, it required manual tuning of model parameters (e.g., seed size in the seed growth segmentation) to achieve desired performance on images collected using different camera systems and/or from different vineyards. This has been the major limiting factor of the method for practical applications. Most research that used DL-based methods investigated deep convolutional neural networks (CNNs) for disease image classification and achieved high classification accuracy (over 97%) of grape DM and PM (Liu et al., 2020; Wang et al., 2021; Suo et al., 2022). A study also reported the use of object detection (e.g., YOLO variants) to detect grape DM infections with a mean average precision (mAP) of 89.55% at an intersection over union (IoU) of 0.5 and processing speed of 58.82 frames per second (FPS) (Zhang et al., 2022). With both high detection accuracy and efficiency, the reported model could be integrated with ground robots for realtime grape DM detection and treatment. From these studies, various attention mechanisms (Niu et al., 2021) and multiscale feature fusion were the two important components contributing to the high model accuracy and generalizability, which should be retained in the future. To the best of our knowledge, our previous study was the only one to examine the use of deep semantic segmentation models for grape DM evaluation in the vineyard (Liu et al., 2021). While the hierarchical multi-scale attention for semantic segmentation (HMASS) model (Tao et al., 2020) was used and evaluated, the performance was obtained only on small training and validation datasets without a separate testing dataset. Furthermore, the entire image-based approach to disease infection quantification should be fully introduced and thoroughly evaluated for both grape DM and PM.

The overarching goal of this study was to develop and evaluate an image-based approach for the quantification of grape foliar disease infection in the vineyard. Grape DM and PM were used as example diseases in this study. Specific objectives were to (1) train and analyze a deep learning model for semantic segmentation of grape DM and PM infections; (2) develop a deep learning-based processing pipeline for disease infection quantification; and (3) evaluate the performance of disease severity quantification by comparing the pipeline-derived and human-assessed measurements.

## 2. Materials and methods

### 2.1. Data acquisition system

**Figure 1:**
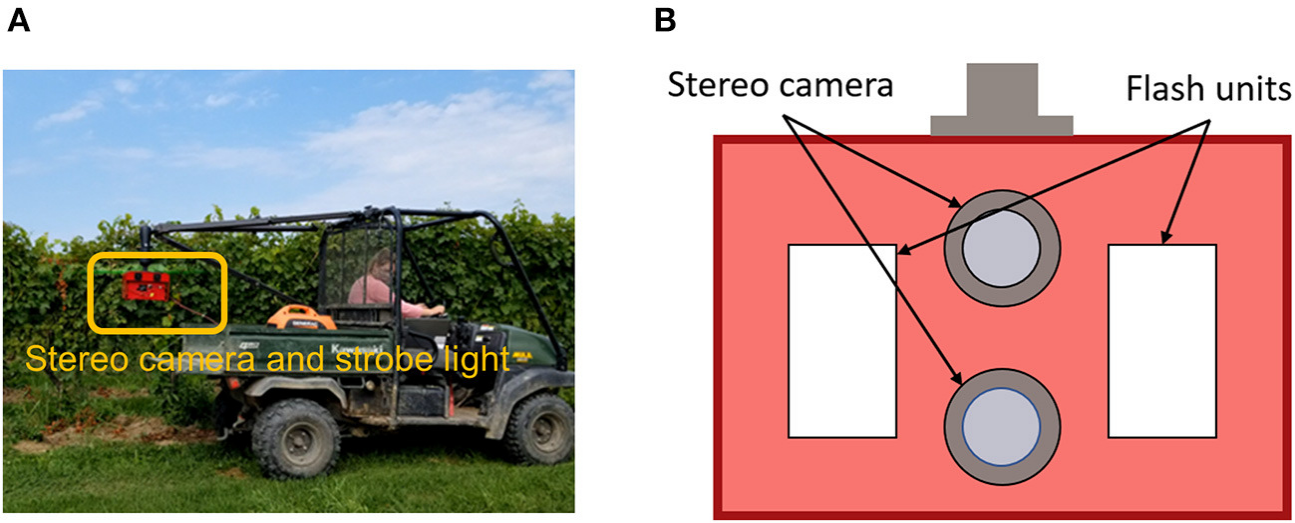
Data collection system used in this study. **(A)** The imaging and illumination module highlighted in the orange box could be flexibly mounted on the ATV. **(B)** A reference detailed design of stereo camera and strobe light system (Silwal et al., 2021).

A data acquisition system (DAQ) was designed to collect color images of grapevines in the vineyard (Figure 1A). The system consisted of a utility task vehicle (UTV) as a mobile platform, a custom stereo camera, a real-time kinematics GPS (RTK-GPS) receiver, and a power generator as power supply for the camera and GPS. The custom camera contained a stereo camera and strobe light illumination units (Figure 1B). The strobe lights were synchronized with the stereo camera shutter, so images were acquired using a fast exposure time (100μs) under a strong flash illumination (Mueller-Sim et al., 2017). This allowed the suppression of irrelevant background information collected in images. The stereo system was configured vertically, so a typical left-right stereo image pair was referred as a top-bottom pair in this study. Image acquisition frequency was set as five frames per second (FPS). During the data collection in a grapevine row, the UTV was driven by cruise control at a speed of approximately 1 m/s for movement consistency. Raw images were stored in portable gray map (PGM) format. A custom program was developed to convert the images to JPG format for processing.

### 2.2. Deep learning-based quantification of grape foliar disease infection

**Figure 2:**
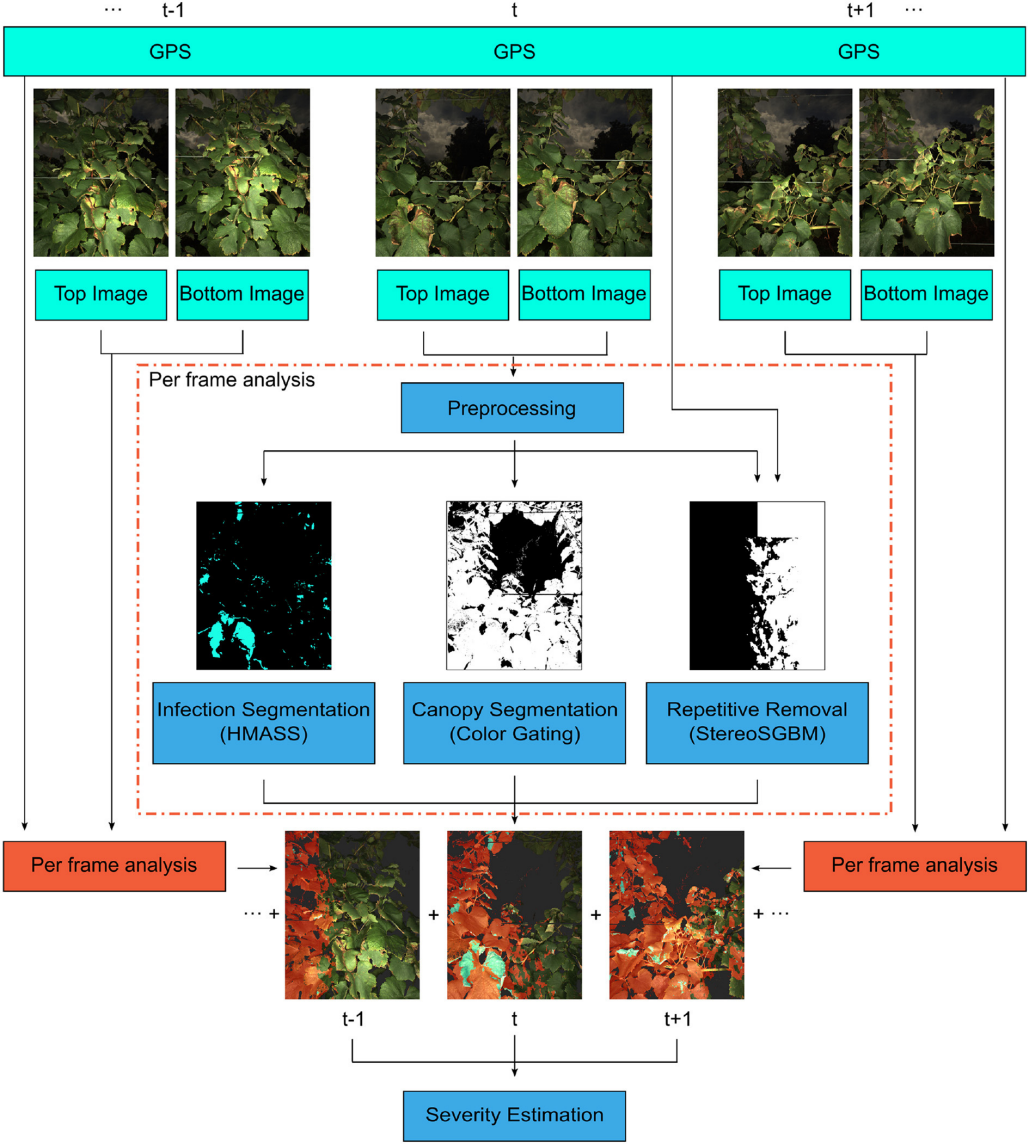
The processing pipeline for disease severity quantification. For each frame, the stereo image pair and GPS location were used to generate pixel-level disease classifications (masks). These masks were combined to provide panel severity rates. Hierarchical Multi-scale Attention for Semantic Segmentation (HMASS) network is a deep convolutional neural network for semantic segmentation.

A deep learning-based processing pipeline was developed to quantify the severity of grape foliar disease infection using a sequence of stereo images (Figure 2). The processing pipeline consisted of four modules: disease infection segmentation, canopy segmentation, image overlap removal, and infection severity estimation. The first three modules were to process individual image pairs in an image sequence, and the last module was for the a given sequence as a whole.

#### 2.2.1. Disease infection segmentation

A deep semantic segmentation model was trained and used for the identification of disease infected regions in images. Foliar disease infections varied dramatically in their sizes because of infection time and progress differences, requiring the multiscale analysis capability of a segmentation model. In this study, the hierarchical multi-scale attention for semantic segmentation (HMASS) network (Tao et al., 2020) was selected because of its flexibility of the network configuration for multiscale analysis at the inference stage without model retraining. The HMASS network learned model weights between two adjacent image scales (e.g., 0.5× and 1× images) during the training phase, and chained the learned weights to combine multiple scales (e.g., adding 0.25× with 0.5× and 2× with 1×) for analysis during the inference phase. Generally, if the ratio between two training scales is *r*, all scales (*s*_*k*_) can be combined for inference if they fulfill the criterion $s_{k}=r^{k},k\in \mathbb{Z}$.

HMASS models were initialized with weights pretrained on the Microsoft Common Objects in Context dataset (Lin et al., 2014) and fine-tuned on the collected grapevine image datasets for disease segmentation. To avoid potential sacrifice of image details caused by image resizing, each original grapevine image (2,704 × 3,376 pixels) was split into 6 subimages (1,352 × 1,125 pixels) as input for processing. Based on our prelimineary tests, image scales of 0.5× and 1× were used for model training, and scales of 0.5×, 1×, and 2× were selected for inference. In addition to common data augmentation practices, class uniform sampling was used to select image regions with balanced class ratios (e.g., ideally the same number of pixels belonging to each class) for model training (Zhu et al., 2019). To ensure full model convergence, the Adam optimizer with an initial learning rate of 5 × 10^−3^ and a batch size of 4 was used to optimize models for 500 epochs. Training, validation, and inference programs were implemented using PyTorch (v1.7.0) and conducted on a server computer with two GPU cards (RTX A6000, Nvidia Corp, Santa Clara, CA). The training environment, parameters, and strategies were the same for all the disease datasets used in this study.

#### 2.2.2. Canopy segmentation

The canopy segmentation module generated grapevine canopy masks in images. The active illumination with a fast camera shutter provided stable lighting intensity of images, enabling the use of simple color filtering for canopy segmentation. An input image was converted from the red, green, blue (RGB) color space into the hue, saturation, value (HSV) space where illumination effects on color appearance could be further isolated. The color filter was designed to identify grapevine canopies and remove irrelevant background such as grass and wooden poles (Equation 1). The filtering thresholds were carefully tuned and verified on representative images in the datasets.

(1) $$\begin{array}{l} \{\begin{array}{l} H_{p}\in \left[12,22\right)\cup \left(28,30\right)\cup \left(45,255\right]\\ S_{p}\in \left[73,153\right)\\ V_{p}\in \left[23,33\right)\cup \left(94,254\right] \end{array} \end{array}$$

where [*H*_*p*_, *S*_*p*_, *V*_*p*_] are the HSV values for each pixel. A pixel was classified as canopy only if that pixel's HSV values satisfied all three conditions. After the color filtering, an open-close operation was applied to fill holes and remove small noise areas in the final canopy mask.

#### 2.2.3. Overlapping area removal

**Figure 3:**
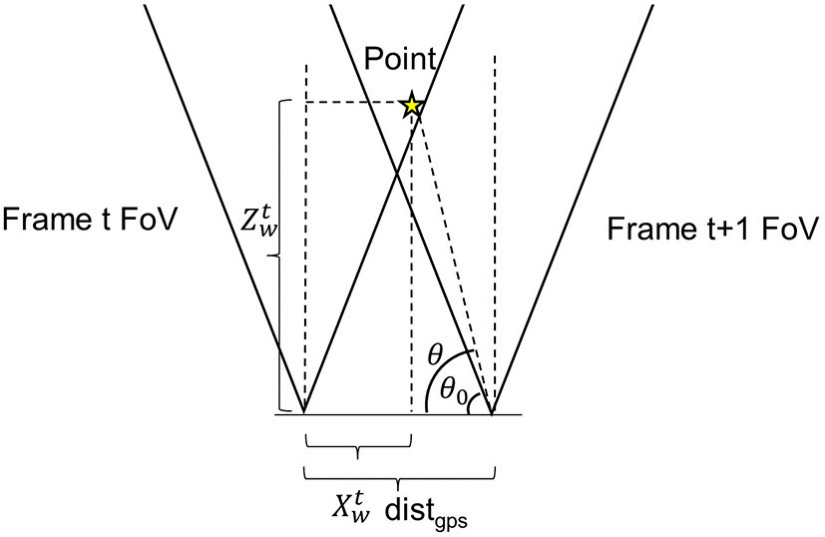
Illustration of the repetitive pixel criterion. θ and θ_0_ were point positioning angle and minimum visible angle. *AoV* is the angle of view of the camera. *x*_*w*_ and *z*_*w*_ were point horizontal and in-depth locations in the world coordinate frame. d was the distance between the adjacent frames. The point would have been marked as repetitive in frame *t* since it falls into the angle of view of camera at frame *t* + 1.

To avoid repeated counts of both grapevine canopy areas and disease infections, the overlapping area removal module used the depth and GPS information to identify and remove repeated regions between consecutive images (Figure 3). The depth information of individual stereo images was obtained using the stereo semi-global block matching (StereoSGBM) method (Hirschmuller, 2007). The custom stereo camera was calibrated to rectify the two images of a stereo pair. Based on preliminary tests, key parameters of the StereoSGBM were set as minimum disparity of 180 pixels, maximum disparity of 356 pixels, and matching window size of 9 pixels. To smooth generated disparity maps, a Gaussian blur operation was applied with a kernel size of 11 x 11 pixels. The resultant disparity maps were converted to depth maps for the projection of 2D image pixels to 3D coordinates (Equations 2–4).

(2) $$\begin{array}{l} z_{w}=\frac{f_{x}*b}{d} \end{array}$$

(3) $$\begin{array}{l} x_{w}=\frac{x_{i}*z_{w}}{f_{x}} \end{array}$$

(4) $$\begin{array}{l} y_{w}=\frac{y_{i}*z_{w}}{f_{y}} \end{array}$$

where *x*_*i*_, *y*_*i*_ were the coordinates of a pixel (*p*) in an image and *x*_*w*_, *y*_*w*_, *z*_*w*_ were the corresponding 3D coordinates of that pixel, *b* was the baseline between the two cameras of a stereo system, *d* was the disparity of *p*, and *f*_*x*_, *f*_*y*_ were the horizontal and vertical focal lengths.

For two consecutive stereo images (i.e., image frames *t* and *t* + 1), the distance difference between their acquisition locations was calculated using their GPS records. As the two images were acquired within a short period of time, the camera could be considered to move primarily along the platform moving direction (the grapevine row direction) with negligible displacement in other directions such as up-and-down movement along y-axis and far-and-close movement along z-axis (across grapevine row). Thus, the overlap region calculation could be simplified using the horizontal (x-axis) camera field of view (FOV). Given the camera angular field of view (AFOV) and depth information, repeatedly imaged regions between the consecutive images were identified using Equation (5).

(5) $$\begin{array}{l} \theta_{0}\leq \theta \Rightarrow \frac{\pi -AFOV}{2}\leq arctan\left(\frac{z_{w}^{t}}{dist_{gps}-x_{w}^{t}}\right) \end{array}$$

where the θ_0_ was the complementary angle of the maximal camera viewing angle, θ was the complementary angle of a given point (*pt*) projected from an image pixel, *dist*_*gps*_ was the distance between the two imaging locations, and $x_{w}^{t}$ and $z_{w}^{t}$ were the 3D x- and z-coordinates of *pt* at the frame *t*. By substituting (Equations 2–5) was reformulated as Equation (6).

(6) $$\begin{array}{l} \frac{f_{x}*f_{y}*b}{dist_{gps}*f_{x}*D-b*f_{y}*X_{i}}-tan\left(\frac{\pi -AFOV}{2}\right)>0 \end{array}$$

where *X*_*i*_, *D* were matrices (*x*_*i*_ ∈ *X*_*c*_ and *d* ∈ *D*) with dimensions identical to image size. During programming, this criterion was transformed to take matrices of associated parameters for all pixels in the image as input and evaluated the repetitiveness at once. As a result, the computational cost was largely reduced. All pixels in the current frame fell into the criterion were marked as repetitive pixels and were not considered in the calculation of infection severity to avoid potential “double-counting” issue.

#### 2.2.4. Infection severity quantification

The severity of disease infections was quantified using the ratio of infected and canopy areas in non-repeated image regions (or effective regions in this study). For each image, the infection and canopy masks in the effective regions were calculated by excluding pixels in the overlapping areas from the masks (Equations 7 and 8).

(7) $$\begin{array}{l} M_{inf,eff}^{i}=M_{inf}^{i}-M_{inf}^{i}\cap M_{rep}^{i} \end{array}$$

(8) $$\begin{array}{l} M_{canopy,eff}^{i}=M_{canopy}^{i}-M_{canopy}^{i}\cap M_{rep}^{i} \end{array}$$

where $M_{inf}^{i}$, $M_{canopy}^{i}$, $M_{rep}^{i}$ represented infection mask, canopy mask, and overlapping area mask of the *i*th image frame, respectively, and $M_{inf,eff}^{i}$, $M_{canopy,eff}^{i}$ represented effective infection and canopy masks of the *i*th image frame, respectively.

Grapevines were trained with a trellis system, so the grapevine canopies would be largely on the same plane. The infection severity could be estimated using image pixels rather than the physical areas in the 3D space. Therefore, for a given grapevine unit (e.g., a panel containing four grapevines), the infection severity (*s*_*zone*_) was calculated by dividing the total effective infection areas (in pixels) by the total effective canopy areas (in pixels) in the image sequence collected in that grapevine unit (Equation 9).

(9) $$\begin{array}{l} s_{zone}=\frac{\sum_{i=0}^{N}\sum M_{inf,eff}^{i}}{\sum_{i=0}^{N}\sum M_{canopy,eff}^{i}} \end{array}$$

### 2.3. Case study demonstration and evaluation

We evaluated the performance of the developed processing pipeline in two case studies on grape DM and PM fungicide efficacy. The two studies were performed at the Cornell Pathology Vineyards of Cornell AgriTech in Geneva, NY in 2019. In the vineyard, grapevine rows 1 to 3 were used for the DM fungicide trials, and rows 6 to 13 were used for the PM fungicide trials.

#### 2.3.1. Downy mildew fungicide efficacy trial

The DM fungicide efficacy study was conducted involving 3 rows of 33-year-old chardonnay vines located on the edge of the field, with each being split into 16 panels of 4 vines each. Two adjacent panels were combined as one spray unit, resulting in a total of 24 spray units. Six treatments were applied to the spray units arranged in a randomized complete block design (RCBD) with 4 replicates per treatment (Supplementary Table S1). A hooded boom sprayer operating at 100 PSI was used to disperse treatments at a volume of 50 gallon per acre (GPA) pre-bloom and 100 GPA post bloom. To perform DM fungicide trials on these rows, additional chemical applications were made to control other grape diseases such as PM and pests.

Field data collection was conducted using the DAQ system on 29 August 2019, and the collected dataset contained 2072 stereo image pairs and their associated GPS locations. The images were segregated into individual panels based on the image content and GPS information. Human field scouting was conducted right after the data collection to provide reference measurements. A human expert sampled and evaluated 20 leaves for each spray unit (i.e., two adjacent panels with the same treatment). DM infection severity was graded as the ratio between infected and total leaf area for each leaf sample based on the Horsfall-Barratt scale (Horsfall and Barratt, 1945). All leaf grades from a spray unit were averaged to calculate the unit DM infection severity, resulting in a total of 24 reference measurements.

To use the developed processing pipeline, an HMASS model was trained using the collected dataset. Two panels that showed considerable infections (treatment 3 and control groups) were randomly selected to provide a total 224 annotated subimages (1,352 × 1,125 pixels) for model training and validation (Table 1). For rigorous model evaluation, additional 463 subimages were selected from a different grapevine row, including panels from treatment 3,4,5, and the control group (Table 1). The testing subimages were processed and annotated in the same way as the training-validation ones.

**Table 1:** Datasets for the segmentation of downy mildew infections.

**Dataset**	**Number of tiles**	**Treatment^a^**	**Panel^b^**
Training	183	3 and control	03–10 and 02–16
Validation	41	3 and control	03–10 and 02–16
Testing	103	3	01–15
	118	4	01–03
	114	5	01–13
	128	Control	01–05

a See Supplementary Table S1 for treatment details.

b Panels are formatted as xx-yy where xx is the row number and yy is the panel number.

#### 2.3.2. Powdery mildew fungicide efficacy trial

The PM fungicide efficacy study included 7 rows of 33-year-old chardonnay vines with the identical layout to the DM trials. The PM fungicide trial rows were 3 rows apart from the DM fungicide trial rows. A total of 18 treatments were applied in the RCBD arrangement with 4 replicates per treatment using the same sprayer system (Supplementary Table S2). To perform PM fungicide trials on these rows, additional chemical applications were made to control other grape diseases including grape DM.

Field image collection was conducted using the same DAQ system on 29 August 2019, followed by human field assessment of PM infection using the Horsfall-Barratt scale and leaf sampling and analysis procedures. Compared with the DM dataset, the PM dataset presented considerable challenges even for experienced human experts to identify PM infections in images. As a result, images collected from only control groups (the most infections) were included in annotation. The dataset contains 132 training tiles, 27 validation tiles, and 16 testing tiles from the PM control group located at panel 13 and 14 of row 10.

### 2.4. Evaluation methods

Performance evaluation was conducted for both individual pipeline components and the full processing pipeline. For the disease segmentation model, visual inspection of representative results and model training and validation curves were used for qualitative evaluation, and the mean intersection over union (mIoU) was used as the metric for quantitative evaluation. The quality of overlapping area removal was also assessed by visual inspection of representative cases because of challenges in ground-truth annotations.

Disease infection severity calculated using the developed pipeline was evaluated in terms of the measurement accuracy and effectiveness in differentiation of treatments. Pearson correlation analyses were conducted between infection severity rates measured by the pipeline and human field assessment. The correlation coefficient (r) was used as a metric to evaluate the goodness of the image-derived measurements. ANOVA analyses with post-hoc Tukey test were performed to differentiate the disease control efficacy among treatments using severity rates calculated by the pipeline and human field assessment. For each measurement method, the severity rates were normalized against the highest measurement value to avoid potential artifacts of ANOVA tests due to value range differences. All analyses and tests were performed using the stats package (v4.0.5) in R, and the significance level of 0.05 was used for the tests unless stated otherwise in the results.

## 3. Result

### 3.1. Downy mildew fungicide efficacy trial

#### 3.1.1. Performance of grape DM segmentation

**Figure 4:**
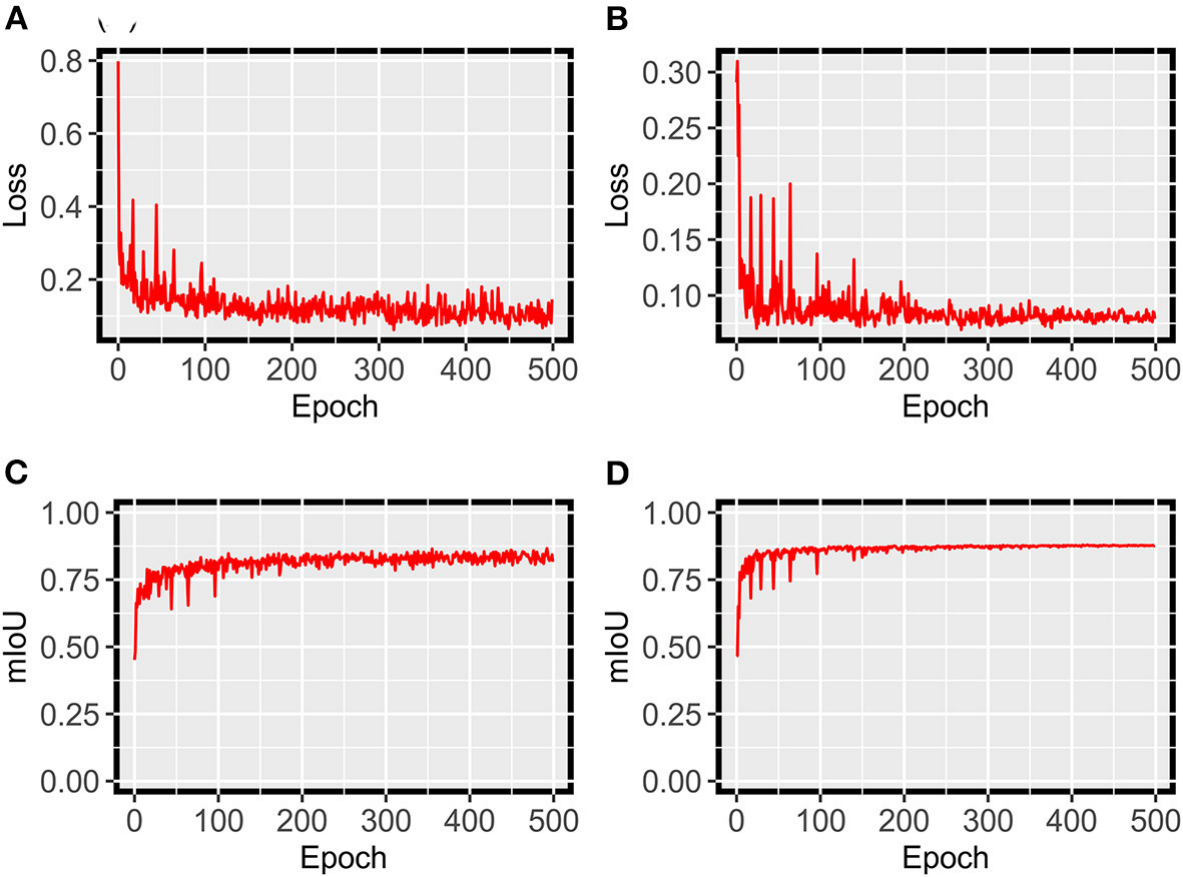
HMASS training and validation process for DM infection segmentation dataset. Parameters were evaluated after each epoch. **(A)** Represents the cross-entropy loss evaluated on the training dataset. **(B)** Represents the cross-entropy loss evaluated on the validation dataset. **(C)** Represents the mIoU evaluated on the training dataset. **(D)** Represents the mIoU evaluated on the validation dataset.

For grape DM segmentation, while a relatively small dataset was used, the HMASS model showed satisfactory training and validation accuracy (Figure 4). During the training process, the model quickly converged in the first 50 epochs and stabilized after 200 epochs, achieving mIoUs of 0.8 and 0.88 for the training and validation datasets, respectively. The high performance was due primarily to two reasons. First, the active illumination of the imaging system minimized the variation among collected images caused by ambient light changes, resulting in a relatively consistent data distribution that would require less training samples for robust performance. This has been investigated as the key for agricultural applications (Silwal et al., 2021). Second, in addition to common data augmentation, the use of class uniform sampling selected image regions with approximately balanced class ratio, enhancing the presence of underrepresented classes (e.g., DM infected areas) for model training. Compared with common image-level augmentation methods, the class uniform sampling augmented at the class level and facilitated the learning of important features for segmenting all classes (Zhu et al., 2019; Tao et al., 2020). Furthermore, the HMASS model achieved an overall mIoU of 0.84 on the testing dataset consisting of more (2.5 and 10 times larger than the training and validation datasets) and unseen (from different grape panels and treatments) images, confirming the high performance during model training and validation.

**Figure 5:**
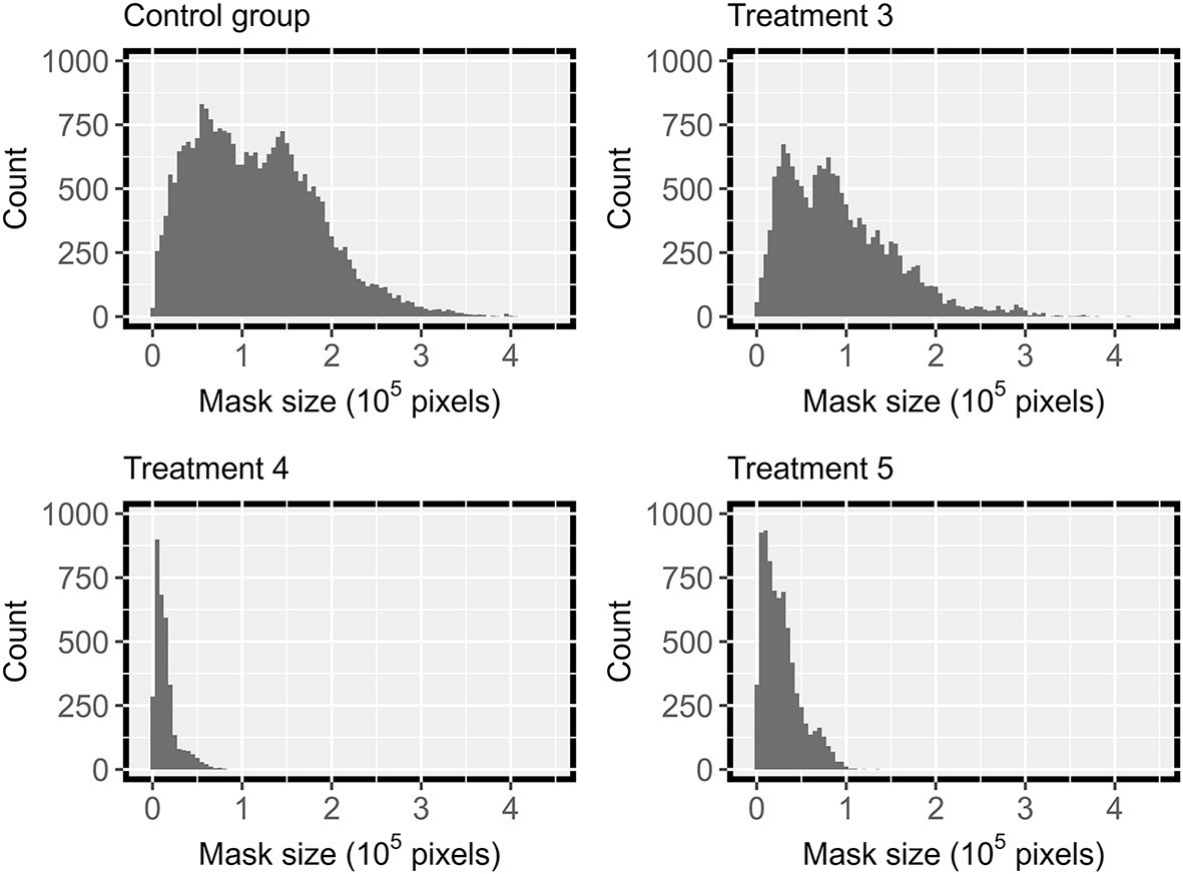
Distribution of manually labeled ground truth mask size in DM testing datasets from treatment 3,4,5 and the control group, with severity rating of of “b”, “bc”, “c”, “a”, in Table 2 respectively.

Since the testing dataset contained images from various treatments, the trained HMASS model was also evaluated using images from individual treatments, and the results showed a performance difference among treatments (Table 2). Severely infected treatments (i.e., treatment 3 and control group) had a relatively higher accuracy than mildly infected treatments (e.g., treatments 4 and 5). This was because the images had different size patterns of DM infected regions among the treatments (Figure 5). Based on human annotation statistics, images collected in severely infected treatments had many large connected infected regions that were typically larger than 1 × 10^5^ pixels, whereas images collected in mildly infected treatments largely contained spotted and small-sized infections, resulting in a common challenge in semantic segmentation of small objects and thus lower segmentation accuracy (Yang et al., 2020). In particular, compared with common objects (e.g., cars) that have shape features, disease infections usually had unpredictable spatial patterns, posing additional difficulties in accurate semantic segmentation. It should be noted that to eliminate potential influence due to training dataset selection, an HMASS model was trained using images of treatments 4 and 5 and tested on images of treatment 3 and control group in the testing dataset. Comparable segmentation accuracy (mIoU of 0.81 and 0.86 for treatment 3 and control group) was achieved, confirming that the infection size difference would be the major factor yielding the performance gap among treatments.

**Table 2:** Per-treatment downy mildew infection segmentation test.

**Treatment^a^**	**Severity level^b^**	**mIoU**
Control	a	0.87
3	b	0.82
4	bc	0.73
5	c	0.76

a Treatments were sorted in top-down order of the expected severity rate from most severe to mild.

b The severity level were evaluated using human scouting data elaborated in Figure 9.

**Figure 6:**
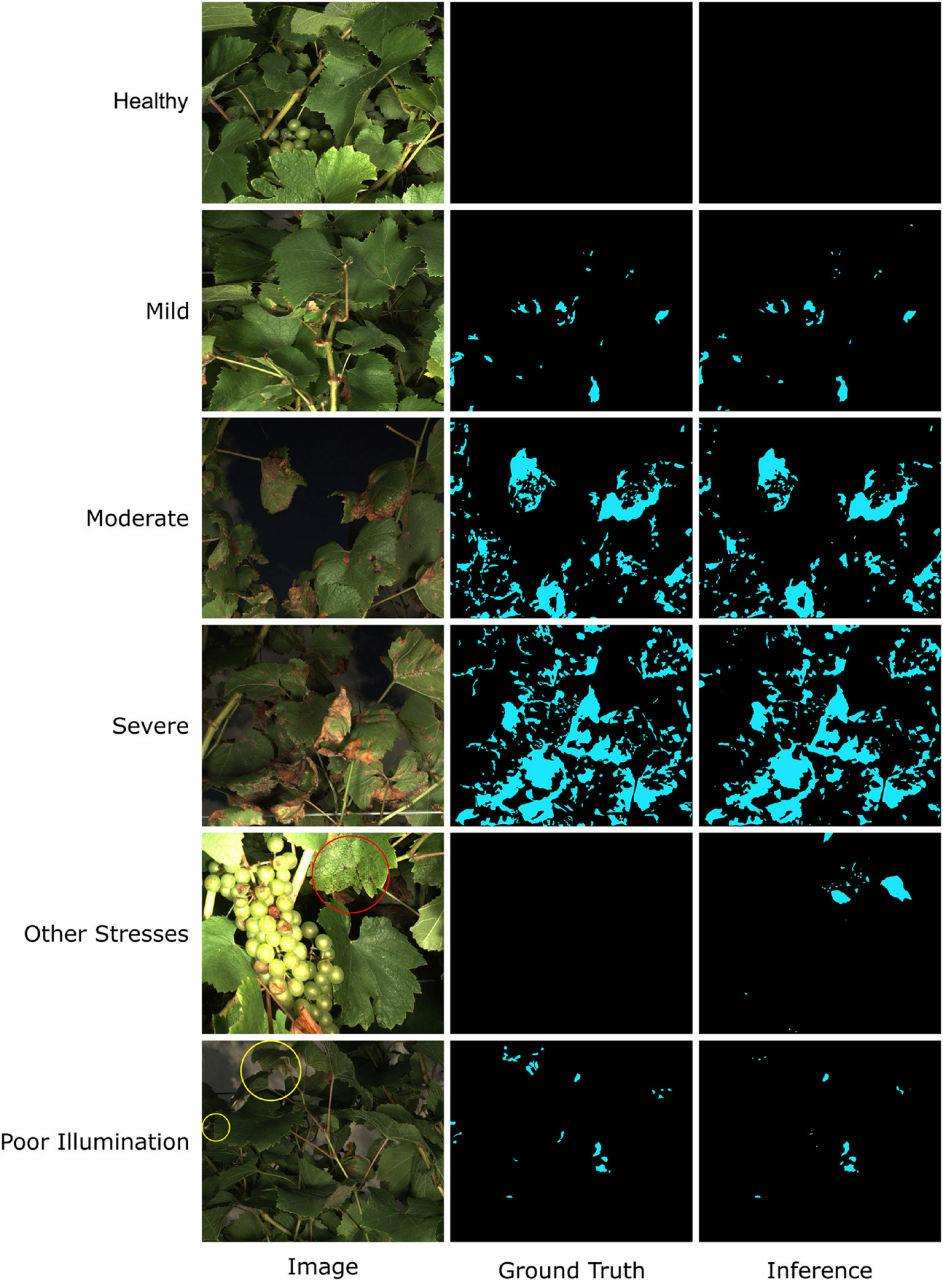
Examples of Downy Mildew (DM) infection segmentation mask generated by HMASS. Images are selected from testing dataset. Ground truth are manually labeled. Red circle indicates region of non-DM damage confused by the inference model. Yellow circles are false negative detections due to insufficient lighting condition.

The model performance was also confirmed by manually checking representative segmentation results (Figure 6). In most cases, the trained HMASS model successfully differentiated healthy and infected (brown regions) canopies and accurately segmented the infected regions with various sizes, which agreed with the high mIoU on the testing dataset (see healthy, mild, moderate, and severe cases in Figure 6). It was noteworthy that compared with the ground truth (human annotation), the model-generated masks might not perfectly detail spotted infections, especially their boundaries and sizes, yielding lower IoU values due to the IoU sensitivity to subtle differences. This would not lead to considerable degradation in disease identification and segmentation. There were some occasions that the trained HMASS model missed or misidentified the DM infections. Some grape leaves presented symptoms that looked similar to grape DM but were caused by other stresses, and the trained HMASS model was not able to correctly identify them (see the red circle in other stresses in Figure 6). In addition, as with all optical systems, the active illumination of the imaging system used in this study had a vignetting effect causing a lower illumination intensity at image corners. Sometimes, the low illumination intensity on an early DM infection (slight leaf discoloration) could reduce the clarity of that infection and miss the segmentation (see the yellow circles in poor illumination in Figure 6).

Although it could not fully address all possible cases, the trained HMASS model generally achieved a satisfactory accuracy of segmenting grape DM and robustly generalized to unseen images. Therefore, it should be sufficiently accurate and robust for grape DM infection identification and therefore quantification in successive steps.

#### 3.1.2. Performance of overlapping region removal

**Figure 7:**
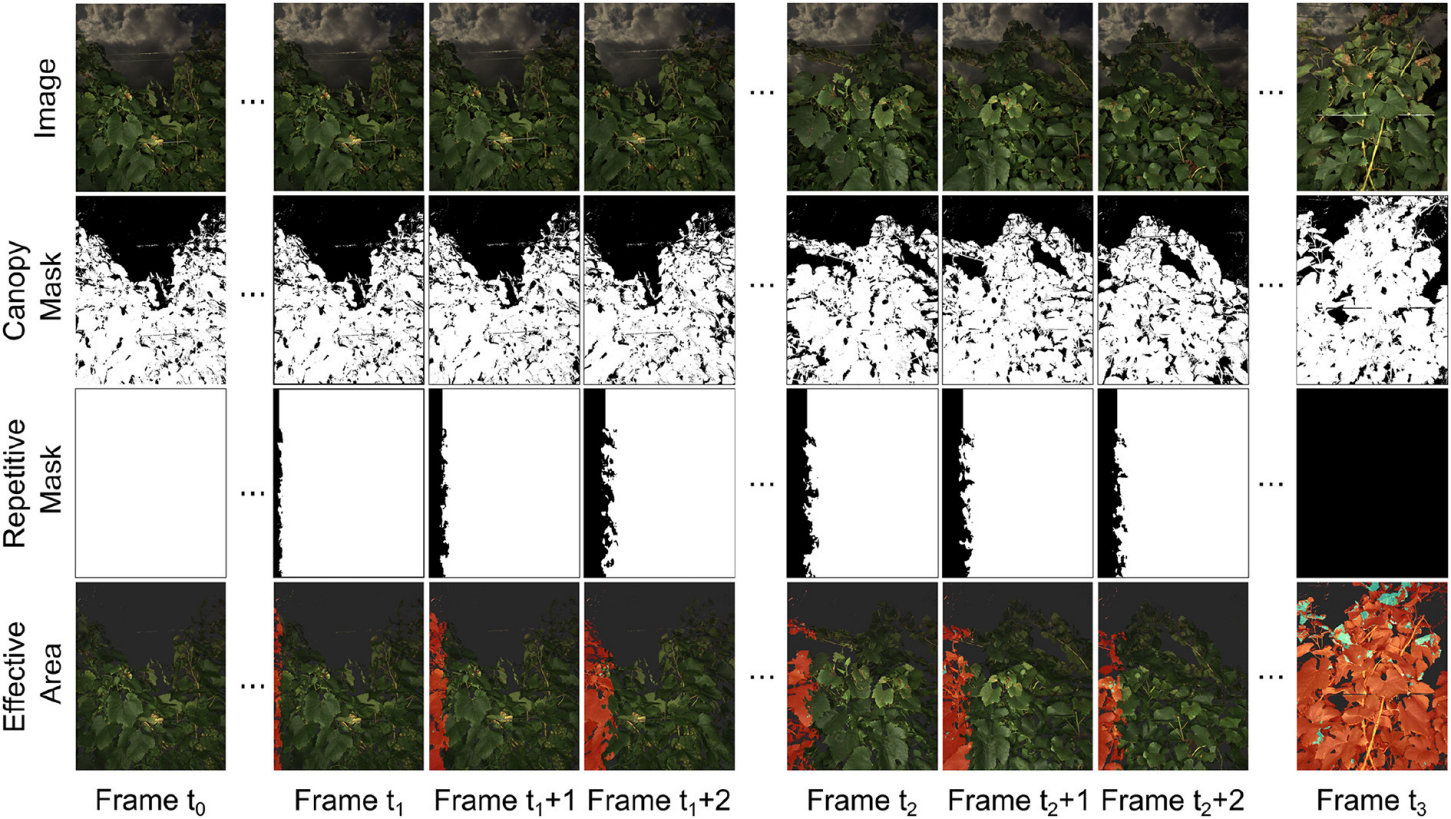
Example masks for repetitive region removal. Data collection vehicle started from stop at *t*_0_ and gradually accelerated from frame *t*_1_ to frame *t*_1_ + 2. The vehicle then moved with constant speed from frame *t*_2_ to *t*_2_ + 2. The vehicle moved out of the data collection region at frame *t*_3_. Canopies areas where the information was kept were labeled in red in effective area images. Infected regions within the effective areas were labeled in blue. The severity rate was determined by the sum of blue areas in the effective area images divided by the sum of red areas in the effective area images.

The overlapping area removal module successfully identified regions that were repeatedly captured between consecutive image frames and provided effective areas for canopy and disease infection quantification (Figure 7). The selected case demonstrated the process for an image sequence collected from an entire grapevine row. At time *t*_0_, the imaging system stopped at the starting point and collected images of the same scene, so the repetitive masks were all white, meaning that all pixels in the current image frame were recaptured in the next image frame and would not be used as an effective area for canopy and infection quantification. From time *t*_1_ to *t*_2_, the imaging system started and accelerated to a preset cruising speed (approximately 1 m/s). During this period, the overlapping areas between consecutive images were reduced, and the non-overlapping areas were identified effective for the quantification and canopy and infection areas. When the imaging system moved at the preset cruising speed (after time *t*_2_), collected image frames showed a relatively constant overlapping region and thus effective area for the quantification. When the imaging system stopped at the ending point (time *t*_3_), the last image frame was considered having no overlapping region and fully used for processing.

Generally, the generated repetitive masks and effective areas were reasonable based on human visual inspection and matched with the imaging system movement patterns (e.g., parked at a point, acceleration, and movement at a constant speed). The effective area in each image frame enabled accurate counting of grapevine canopy and disease infection pixels to avoid the "double-counting" issue. It should be noted that the developed overlapping area removal module was based on the depth and GPS information without the consideration of image features. As a result, complex object geometry might lead to some errors of overlapping region identification, especially near the separation line between overlapped and non-overlapped areas. Also, if the field terrain was considerably uneven (e.g., big bumps and ditches), the imaging system could not be considered moving only along the row direction (i.e., x-axis of the camera), causing additional errors in the generated repetitive masks and effective areas. However, these error sources were either trivial (only a small portion of pixels) or occasional (few big bumps in the field), and therefore, the generated repetitive masks and effective areas were sufficient to resolve potential “double-counting” issues that would considerably affect the accuracy of canopy and disease infection quantification.

#### 3.1.3. Severity rate estimation evaluation

**Figure 8:**
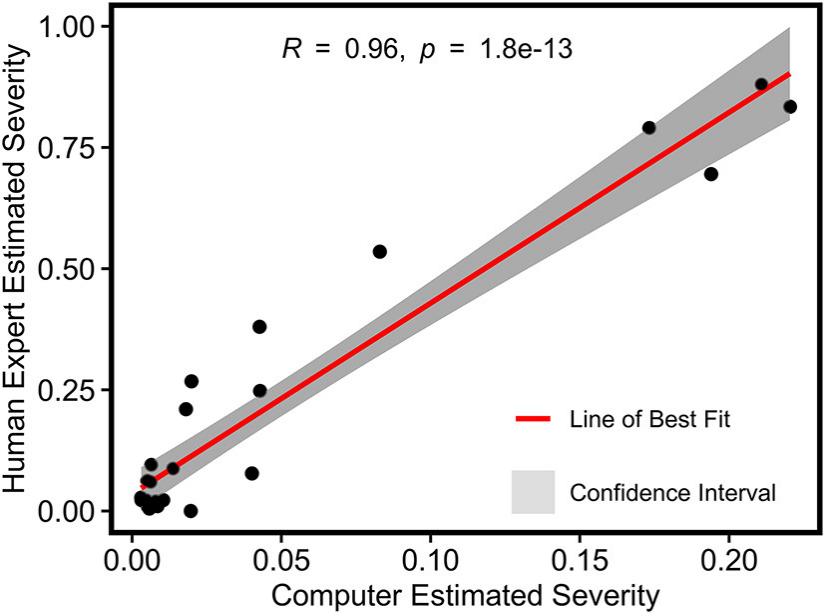
Pearson's correlation result of normalized severity rates of DM fungicide efficacy test calculated using the imaging-based pipeline and human field assessment.

For grape DM infection quantification, there was a strong Pearson's correlation (r = 0.96) between image-derived and human-assessed infection severity rates, indicating the high accuracy of disease infection quantification using the developed processing pipeline (Figure 8). While the theoretical range of image-derived and human-assessed measurements was from 0 (not infected) to 1 (fully infected), severity rates calculated by the two methods showed different magnitudes because of sampling object difference. The human assessment was for individual leaf samples where full infection could occur, whereas the image-based method evaluated the whole grapevine in a specific unit (e.g., a panel) that would not be fully infected in practice. To avoid potential artifacts in successive statistical analyses, the image-derived severity rates were normalized to have the least infection of 0 and the most infection of 1.

**Figure 9:**
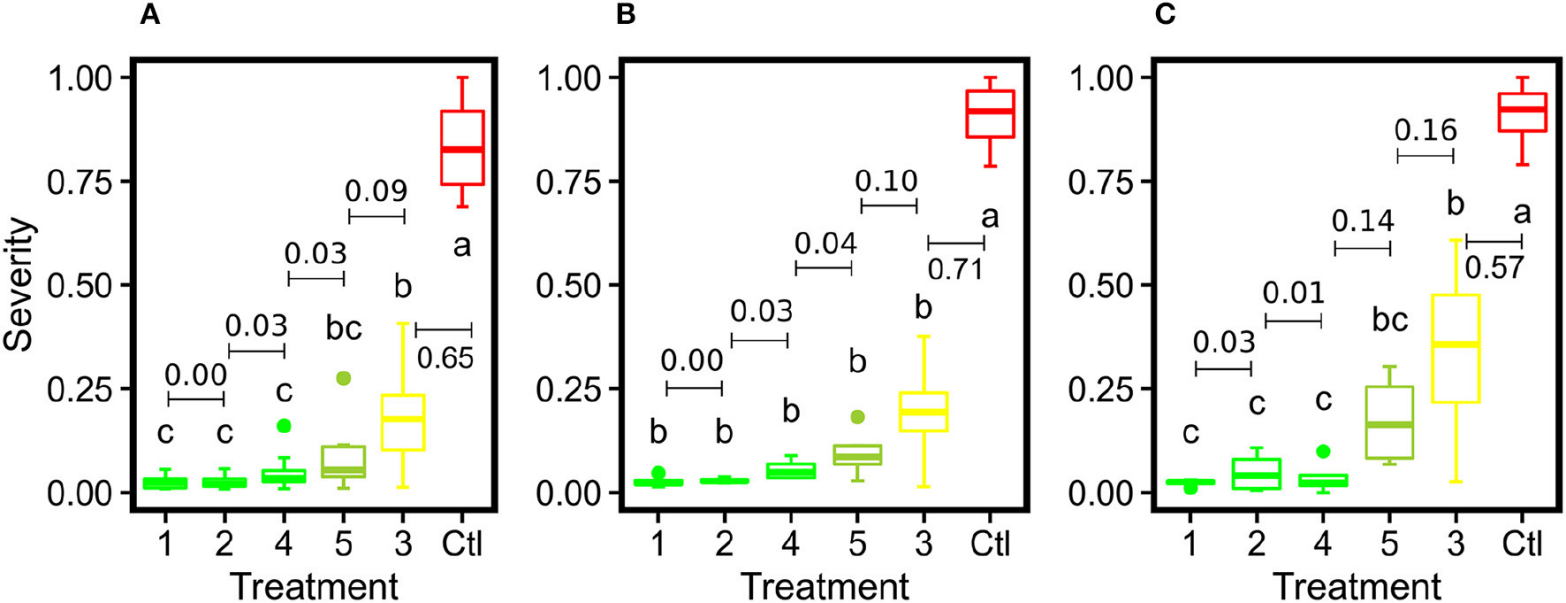
Box plots of severity rates for the six treatments together with the control group for DM fungicide efficacy test in this study: **(A)** represents the imaging-derived severity rates at the panel level, **(B)** represents the imaging-derived severity rates at the spray unit level, and **(C)** represents the severity rates evaluated by human experts. Different letters indicate statistical significance between groups. Treatments within the same group are assigned the same color. The numbers with bars indicate differences of mean values between adjacent treatments.

Generally, the image-derived and human-assessed severity rates showed similar statistical patterns to differentiate grape DM fungicide treatments (Figure 9). When the grape panel was used as the unit, the image-derived severity rates demonstrated an identical statistical pattern as the human-assessed measurements, presenting the effectiveness of using the image-based method to quantify disease infection and evaluate fungicide efficacy. Also, the image-derived measurements showed smaller within treatment variance than the human assessments because of the objective, full panel evaluation over the subjective sampling-based evaluation. When the spray unit (two grape panels) was used, the image-derived severity rates were only capable of distinguishing treatments with considerable differences such as severely infected (e.g., control) and well-controlled groups (e.g., treatment 1). No statistical difference was found between well-controlled to mildly infected treatments. This occurred primarily because of the replication size reduction. The human field scouting collected 20 leaf samples in each spray unit, resulting in 80 (20 × 4 spray units per treatment) replications per treatment. In contrast, the image-based method used each spray unit as a replication and only had 4 replications per treatment. The considerable replication decrease would lead to the substantial reduction of statistical power to differentiate treatments irrespective of the measurement accuracy. In this study, a minimum of 8 replications would be needed, or replicate panel images would need to be split into subsampled images. The optimal replication size requires more investigations in future studies.

### 3.2. Powdery mildew case study

#### 3.2.1. Performance of grape PM segmentation

**Figure 10:**
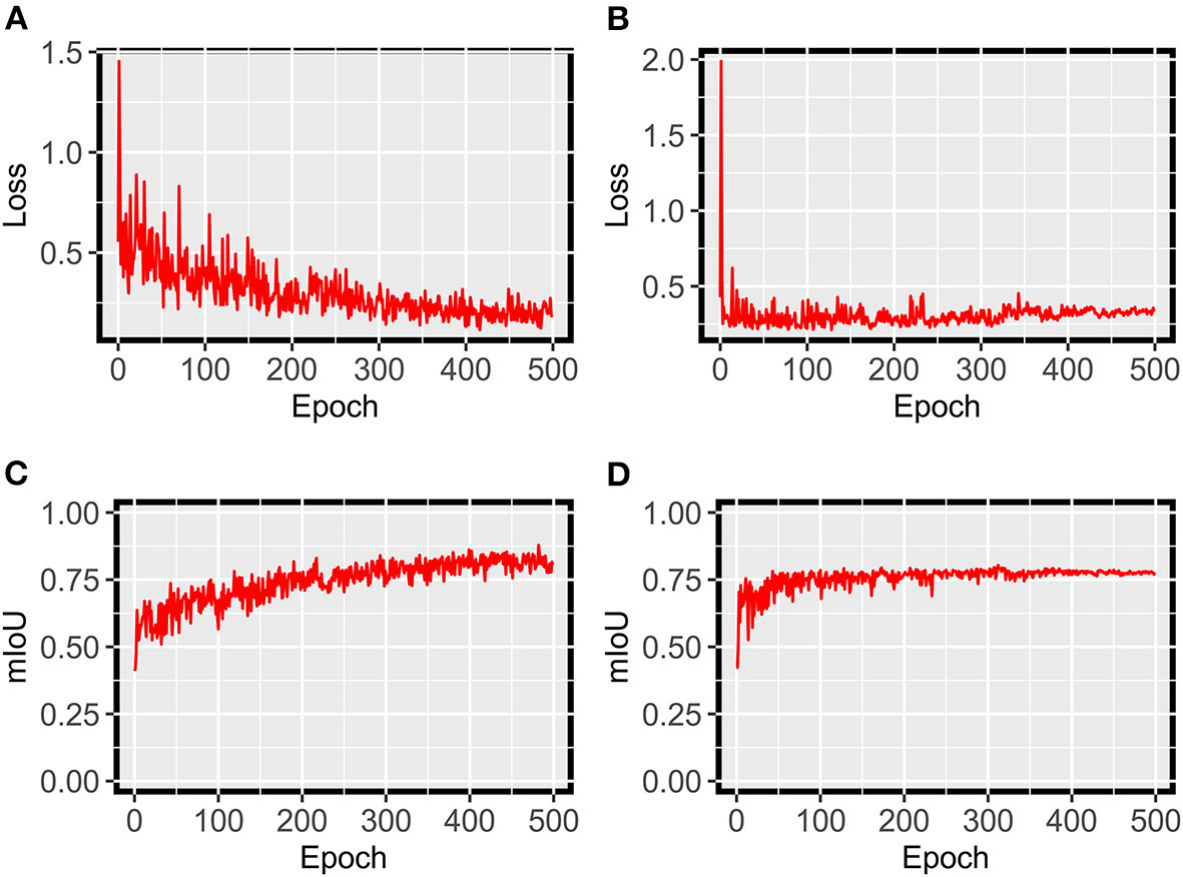
HMASS training and validation process for PM infection segmentation dataset. Parameters were evaluated after each epoch. **(A)** Represents the cross-entropy loss evaluated on the training dataset. **(B)** Represents the cross-entropy loss evaluated on the validation dataset. **(C)** Represents the mIoU evaluated on the training dataset. **(D)** Represents the mIoU evaluated on the validation dataset.

For grape PM segmentation, the HMASS model also converged successfully and showed an acceptable segmentation performance on the training and validation datasets (Figure 10). Compared with the grape DM model, the grape PM model used more epochs (300 epochs vs. 200 epochs) to converge with a relatively lower segmentation accuracy (mIoU of 0.76 for both training and validation datasets). This was mainly because it was more challenging to segment grape PM infections than grape DM infections. Grape DM infections had an obvious discoloration (yellowish to brownish color) that was well captured by the imaging system, whereas grape PM infections only showed the white to gray powdery appearance when they were imaged or viewed from acute angles (optimally 30 to 50 degrees from the leaf surface Oberti et al., 2014), presenting difficulties to not only the model for segmentation but also human experts for annotation. In terms of the model generalizability, the grape PM model demonstrated a good performance (mIoU of 0.73) on the testing dataset containing unseen images. Considering these factors, the trained HMASS model also provided acceptable accuracy for grape PM identification and segmentation to be used for infection quantification.

**Figure 11:**
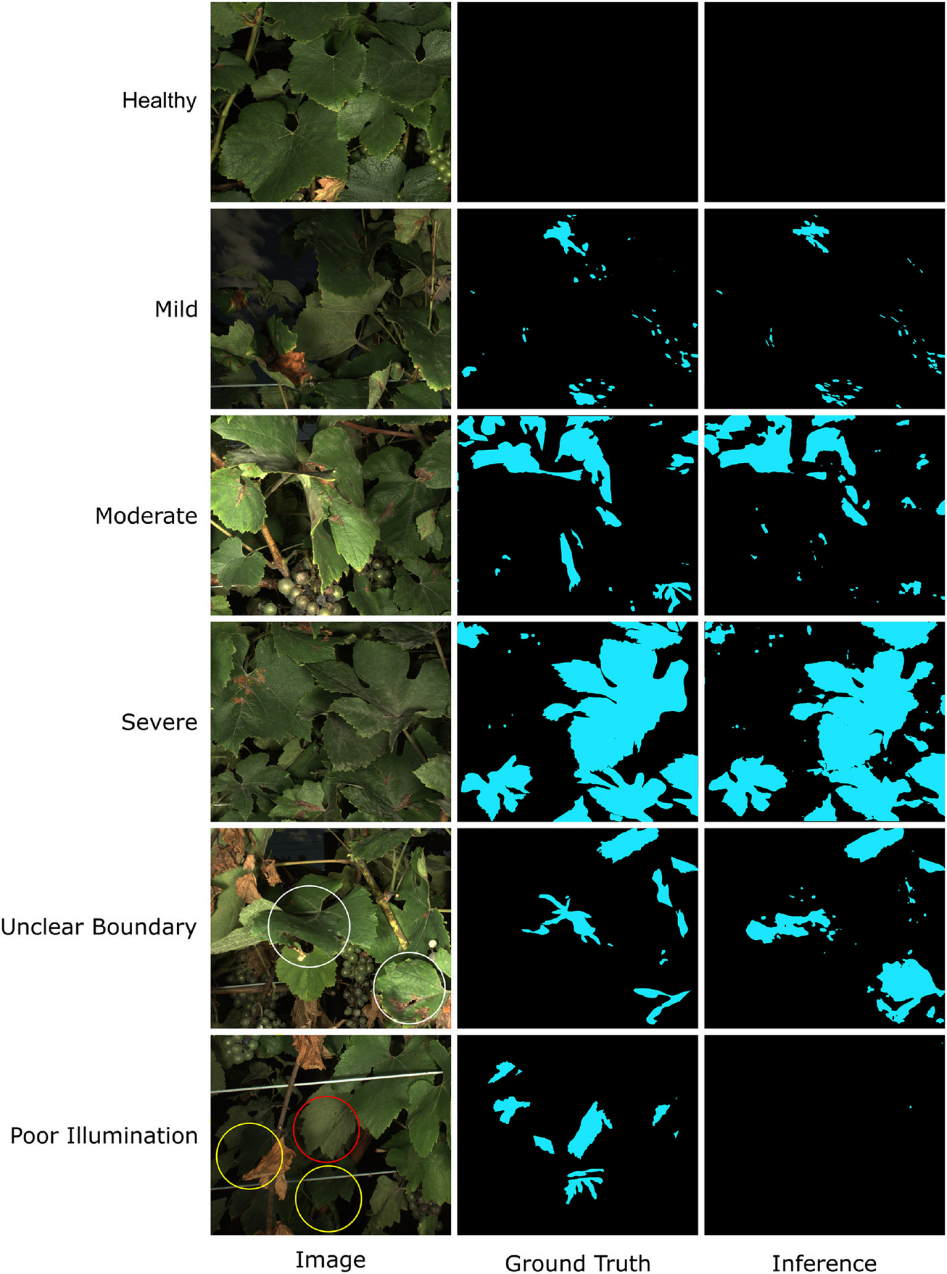
Examples of Powdery Mildew (PM) infection segmentation mask generated by HMASS. Images are selected from testing dataset. Ground truth are manually labeled. White circles are regions where boundaries of infections are not correctly detected. Yellow circles are false negative detections due to insufficient lighting condition. Red circles are false negative detection due to variant unobvious appearance of PM damage.

The visual inspection of segmentation results of testing images confirmed the training performance evaluation (Figure 11). In most cases, the trained HMASS model successfully identified and segmented PM infections with varying sizes and infection severity, showing the model effectiveness and generalizability (see healthy to severe cases in Figure 11). Similarly to the grape DM segmentation, spotted and small-sized infections were the challenging cases and showed imperfect segmentation details (e.g., some spotted infections might be missed). A major challenge to the PM segmentation was the inadequate capability of sensing all PM infections with complex leaf geometry and orientation. If a leaf was imaged with its surface parallel with the imaging system, the leaf surface details were considerably reduced, resulting in the lack of texture and color features to delineate PM infections. As a result, the HMASS model could not reliably segment the infection boundary (see white circles in the unclear boundary case in Figure 11). The visibility of PM infections also substantially decreased in some cases where leaves were underlit (see yellow circles in the poor illumination case in Figure 11) and/or totally perpendicular to the camera (see the red circle in the poor illumination case in Figure 11), leading to the false negative identification of those regions (i.e., miss the infections). Since these extreme cases represented a small portion of all possible leaf orientation and geometry and limitations were mainly from the sensing system, the trained HMASS model performance was sufficiently accurate to identify and segment PM infections with clear symptoms for successive analyses.

#### 3.2.2. Severity rate estimation evaluation

**Figure 12:**
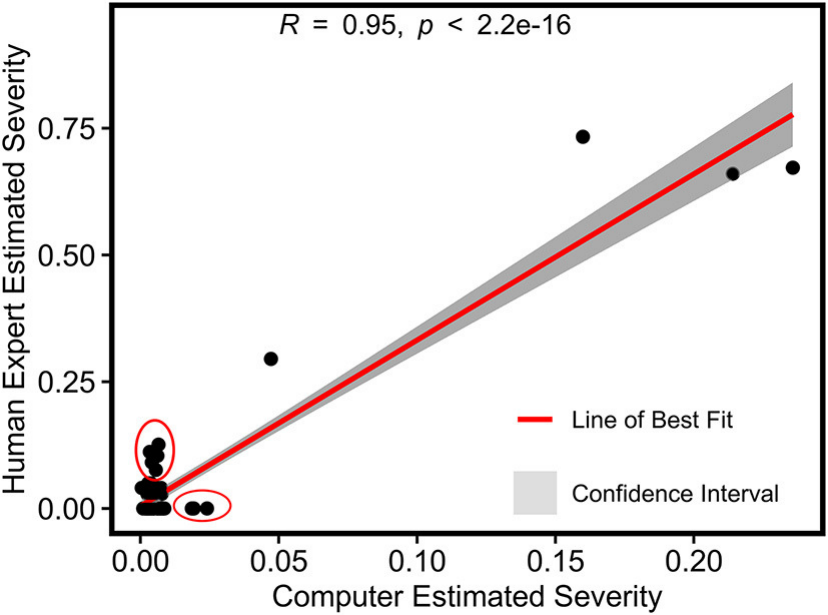
Pearson's correlation result of normalized severity rates of PM fungicide efficacy test calculated using the imaging-based pipeline and human field assessment. Red circles represent outliers in the mildly infected cases.

A strong Pearson's correlation (*r* = 0.95) was also found between the image-derived and human-assessed severity rates for grape PM infection (Figure 12). Similarly to the grape DM case study, the image-based method evaluated the entire grape panel or spray unit rather than individual leaf samples, so measurements of the two methods were in different magnitudes. The image-derived severity rate theoretically had the range from 0 (not infected) to 1 (fully infected). Although the overall correlation was strong, many data points representing mildly infected panels showed certain deviations between the image-derived and human-assessed measurements (see the red circles in Figure 12). This was very likely due to the sensing system incapable of resolving mild PM infections in images. Consequently, these infections could be missed by the trained model, introducing errors in the calculation of infection severity rate. As the mildly infected panels had a relatively low absolute severity rate, such errors (mostly underestimation) presented more evident relative effects.

**Figure 13:**
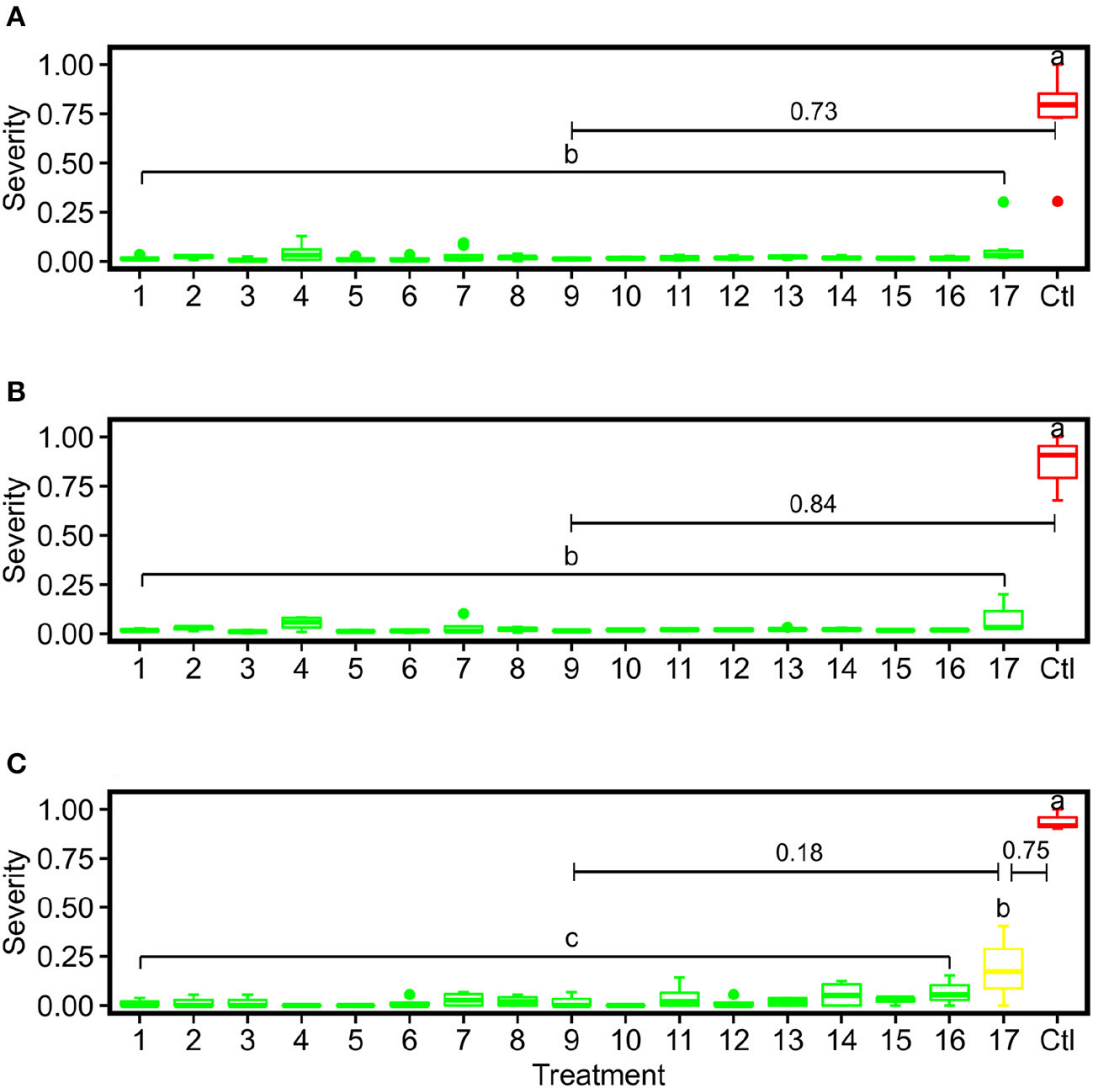
Box plots of severity rates for the 17 treatments together with the control group for PM fungicide efficacy test in this study: **(A)** represents the imaging-derived severity rates at the panel level, **(B)** represents the imaging-derived severity rates at the spray unit level, and **(C)** represents the severity rates evaluated by human experts. Different letters indicate statistical significance between groups. Treatments within the same group are assigned the same color. The numbers with bars indicate differences of mean values between adjacent severity groups.

ANOVA followed by Tukey test results showed that the control group was statistically different from all PM fungicide treatment groups using the severity rates calculated by the image-based method and human field assessment (Figure 13). Although the mean value of treatment 17 was higher than other fungicide treatments, the image-derived severity rates were not able to statistically differentiate treatment 17 from other treatments irrespective of replication unit used (either panel or spray unit), which was the major difference in statistical power than the human-assessed values. Possible reasons causing this were complex. On one hand, the imaging system for entire panel evaluation without subjective leaf sampling should be objective and yield improved severity rate calculation than human field scouting. On the other hand, human field scouting was able to check leaf samples from all possible viewing angles to not miss even mild PM infections, which should be more accurate for disease identification. Thus, it is worthy of further investigations in the future to reveal details. Nonetheless, the objective, full panel evaluation through the image-based method still provided smaller variations within treatment, which would be desired for statistical analyses with limited replications.

## 4. Discussion

The developed image-based approach has demonstrated a high accuracy of the quantification of grape DM and PM infection in the vineyard, enabling high throughput plant disease sensing for fungicide or biocontrol efficacy trials, genetic mapping of disease resistance, and resistance breeding and selection. The high accuracy has been achieved because of both sensing and analytics improvements. From the sensing perspective, the strobe light-enhanced imaging system provides an active and stable illumination to minimize image quality variations caused by ambient light changes, especially in the field. The improved consistency of image quality (mostly illumination-related) allows the use of simple image features (e.g., color) and operations (e.g., filtering) for reliable analyses such as canopy segmentation. Meanwhile, such improved image consistency simplifies the training requirements (e.g., the number of annotated training samples) of modern deep neural networks for downstream tasks such as grape DM and PM segmentation, which agrees with previous studies (Silwal et al., 2021).

From the analytical perspective, the experimental results have agreed with previous studies: modern deep learning models designed for general computer vision tasks can be effectively used for plant stress identification and segmentation with limited training datasets (Singh et al., 2018; Jiang and Li, 2020). These deep learning models provide accurate and implementable solutions to the core needs of plant disease (or stress in general) phenotyping. In addition to this, the developed pipeline has shown the advantage of combining multimodal sensing data for disease quantification. By combining the color, depth (retrieved via stereo camera), and localization information, the developed pipeline is capable of not only segmenting grape disease infections in a single image but also quantifying infection severity in an image sequence without “double-counting” concerns. This can provide a more accurate evaluation of disease infection severity in the field, especially production systems. Since the developed approach and pipeline showed high accuracy of diseases with different symptoms, the approach and pipeline could be potentially expanded to general foliar diseases with visible symptoms, which is substantially beneficial for grape disease research, breeding, and management.

Several key technical limitations have been identified in this study as well. First, the sensing system is the fundamental limiting factor for plant disease detection and quantification. When disease symptoms could not be fully and/or consistently sensed, there might not be too much to improve from data analytics. In this study, even the same deep learning model was used, the mIoU of segmenting grape DM and PM could vary up to 0.11, which is a considerable performance difference. Additionally, although DM and PM cause infections on both sides of the leaf surface and the symptoms on lower leaf surface are usually more obvious, the current imaging system only primarily sense the upper leaf surface due to occlusions. To solve this, one option is to use advanced sensing modalities (e.g., thermal, multispectral or hyperspectral imaging and advanced sensor mounting system), and another option is to optimize sensing systems for specific disease or stress of interest. For instance, the optimal imaging angle of grape PM is from 30 to 50 degrees with the leaf surface, which can guide the design of new optical sensors. Second, plant disease datasets can have some unique challenges to current computer vision (CV) algorithms and models. For instance, both grape DM and PM images have small and scattered infections, especially for mild infection cases. Segmenting these small objects has been widely acknowledged as a challenge even by the CV community (Yang et al., 2020). Plus, compared with common CV datasets, these small disease infections have unpredictable shapes, presenting additional difficulties. In some cases, the infection area may appear non-typical and can look similar to other leaf stresses. Due to the dataset and model limitation, the current method was only developed for typical infections seen in the field of experiments and labeled by human expertise and would not be directly applied for infections of significantly different appearances without further calibration. It requires interdisciplinary efforts on developing new CV models and incorporating domain knowledge to lift the constraints of model and dataset and further improve the data analysis accuracy. Third, experiments conducted in this study were well designed and controlled to allow either DM or PM (not both) to occur in the vineyard, so the collected datasets and trained models were not for the differentiation between multiple diseases. While the presence of a specific disease (or plant stress in general) could be well controlled in research and breeding, production vineyards usually show multiple diseases (even a combination of abiotic and biotic stresses) at the same time. Stress differentiation was not the objective of the present study, but it should be further investigated in the future to maximize the use of digital tools for production systems. Last, the developed pipeline is for offline analysis, which cannot be directly integrated with robots and other machinery for realtime disease quantification. This limits the potential of using the developed technology for vineyard management such as precision spraying. The most time-consuming module is the HMASS-based disease segmentation, so it is necessary to explore options to replace the HMASS model with light-weight ones or to optimize (e.g., model pruning) the HMASS model for online processing.

## Conclusion

An image-based approach was developed for the quantification of grape foliar disease in the vineyard, and evaluated using grape DM and PM fungicide trials. For both diseases, the image-derived infection severity was highly correlated (*r* > 0.95) with human field assessment, and showed effective statistical power in differentiating the efficacy of fungicide treatments. Therefore, the developed approach can be used as an effective tool for grape DM and PM evaluation in research projects and production management. Future studies will be focusing on (1) exploring various sensing modalities for grape PM identification to improve the quantification accuracy and (2) investigating light-weight deep learning models for online disease segmentation and quantification.

## Data availability statement

Raw data supporting the conclusions in this article will be made available by the authors upon requests for research purposes.

## Author contributions

YJ conceived the general idea and provided directions for the pipeline development. EL developed the pipeline and analyzed data. KG and LC-D coordinated the fungicide efficacy trials. KG and DC coordinated field human scouting and dataset annotation. EL and YJ drafted the manuscript. All authors coordinated the imaging and read, revised, and approved the manuscript for submission.

## Funding

This study was supported by the Cornell AgriTech Venture Fund, Cornell Institute for Digital Agriculture Research Innovation Fund, the New York Wine and Grape Foundation, and USDA NIFA Hatch (Accession Nos. 1025032, 1024574). Image data collection and curation were funded by the USDA NIFA Specialty Crop Research Initiative (Award No. 2017-51181-26829).

## Conflict of interest

The authors declare that the research was conducted in the absence of any commercial or financial relationships that could be construed as a potential conflict of interest.

## Publisher's note

All claims expressed in this article are solely those of the authors and do not necessarily represent those of their affiliated organizations, or those of the publisher, the editors and the reviewers. Any product that may be evaluated in this article, or claim that may be made by its manufacturer, is not guaranteed or endorsed by the publisher.
