# Supplementary material for: Deep semantic segmentation for the quantification of grape foliar diseases in the vineyard
Source: Front Plant Sci. 2022 Sep 9;13:978761. doi: 10.3389/fpls.2022.978761 (PMC9501698; doi:10.3389/fpls.2022.978761)
Supplement: Supplementary file 1 [file Data_Sheet_1.pdf]

Supplementary Material

1 SUPPLEMENTARY TABLES

Table S1. Summary of DM fungicide treatments tested in this study[a]

Treatment	Material and rate per acre	Timing (2019)
1	Zampro 14.0 oz[b]	28 May, 11 Jun, 25 Jun, 8 Jul, 23 Jul, 5 Aug, 20 Aug
2	Revus Top 7.0 oz[c]	28 May, 11 Jun, 25 Jun, 8 Jul, 23 Jul, 5 Aug, 20 Aug
3	Lifegard WG 2.25oz[c]	28 May, 25 Jun
	Lifegard WG 4.5 oz[c]	25 Jun, 8 Jul, 23 Jul, 5 Aug, 20 Aug
4	Zampro 14.0 oz[b]	28 May, 25 Jun, 23 Jul
	Lifegard WG 2.25 oz[c]	11 Jun
	Lifegard WG 4.5 oz[c]	8 Jul, 5 Aug, 20 Aug
5	Dithane DF[c]	28 May, 11 Jun, 25 Jun
	Lifegard WG 4.5 oz[c]	8 Jul, 23 Jul, 5 Aug, 20 Aug
6	Untreated control (UTC)	N/A

a. The following applications were also made for all rows in the trial to control against PM and the insect species grape berry moth and Japanese beetle; 3 Jun - Quintec 4.0 oz/A; 17 Jun - Mettle 125ME 5.0 oz/A; 1 Jul - Quintec 4.0 oz/A; 12 Jul - Mettle 125ME 5.0 oz/A and Leverage 360 4.0 oz/A; 24 Jul – Vivando 2.5SC 12.0 oz/A and Assail 30SG 2.5 oz/A; 6 Aug –Vivando 2.5SC 12.0 oz/A.
b. "Silwett L-77" surfactant included in spray solution at 0.03% (v/v) concentration.
c. "Induce" surfactant included in spray solution at 0.125% (v/v) concentration.

Table S2. Summary of PM fungicide treatments tested in this study[a]

Treatment	Material and rate per acre	Timing (2019)
1	VJR84-R0002 6.0 oz[b]	29 May, 11 Jun, 25 Jun, 8 Jul, 23 Jul, 5 Aug, 20 Aug
2	Rhyme 2.08EC 5.0 oz[b]	29 May, 5 Aug
	VJR84-R0002 6.0 oz[b]	11 Jun, 8 Jul
	Vivando 300 SC 10.0 oz[c]	25 Jun
	Torino 0.85 EC 3.4 oz[b]	23 Jul
	Microthiol 5.0 lb[d]	20 Aug
3	Rhyme 2.08EC 5.0 oz[c]	29 May, 11 Jun, 25 Jun, 8 Jul, 23 Jul, 5 Aug, 20 Aug
4	Revus Top 7.0 oz[c]	29 May, 11 Jun, 25 Jun, 8 Jul, 23 Jul, 5 Aug, 20 Aug
5	Revysol 5.0 oz[c]	29 May, 11 Jun, 25 Jun, 8 Jul, 23 Jul, 5 Aug, 20 Aug
6	Prolivio 4.0 oz[c]	29 May, 11 Jun, 25 Jun, 8 Jul, 23 Jul, 5 Aug, 20 Aug
7	Prolivio 5.0 oz[c]	29 May, 11 Jun, 25 Jun, 8 Jul, 23 Jul, 5 Aug, 20 Aug
8	Vivando 300 SC 10.0 oz[c]	29 May, 11 Jun, 25 Jun, 8 Jul, 23 Jul, 5 Aug, 20 Aug
9	Gatten EC 6.0 oz[c]	29 May, 11 Jun, 25 Jun, 8 Jul, 23 Jul, 5 Aug, 20 Aug
10	Luna Experience 6.0 oz[c]	29 May, 11 Jun, 25 Jun, 8 Jul, 23 Jul, 5 Aug, 20 Aug
11	Fervent 475SC 8.5 oz[c]	29 May, 11 Jun, 25 Jun, 8 Jul, 23 Jul, 5 Aug, 20 Aug
12	Pyrazaflumid 3.1 oz[c]	29 May, 11 Jun, 25 Jun, 8 Jul, 23 Jul, 5 Aug, 20 Aug
13	Kenja 15.5 oz[c]	29 May, 11 Jun, 25 Jun, 8 Jul, 23 Jul, 5 Aug, 20 Aug
14	Revus Top 7.0 oz[c]	29 May
	Vivando 300 SC 10.0 oz[c]	11 Jun
	Luna Experience 6.0 oz[c]	25 Jun
	Gatten EC 6.0 oz[c]	8 Jul
	Microthiol 5.0 lb[c]	23 Jul, 5 Aug, 20 Aug
15	Revus Top 7.0 oz[c]	29 May
	Vivando 300 SC 10.0 oz[c]	11 Jun
	Luna Experience 6.0 oz[c]	25 Jun
	Gatten EC 6.0 oz[c]	8 Jul
	Lifegard WG 4.5 oz[c]	23 Jul, 5 Aug, 20 Aug
16	JMS Stylet Oil 1.5%	29 May, 23 Jul, 5 Aug, 20 Aug
	Vivando 300 SC 10.0 oz[c]	11 Jun
	Luna Experience 6.0 oz[c]	25 Jun
	Gatten EC 6.0 oz[c]	8 Jul
17	Lifegard WG 4.5 oz[c]	29 May, 11 Jun, 25 Jun, 8 Jul, 23 Jul, 5 Aug, 20 Aug
18	Untreated control (UTC)	N/A

a. The following applications were also made for all rows in the trial to control against DM and several insect species including Japanese beetle and grape berry moth ; 3 Jun – Phostrol 2.5 pt/A; 17 Jun – Zampro 4.4SC 14.0 oz/A; 1 Jul – Phostrol 2.5 pt/A; 12 Jul – Zampro 4.4SC 14.0 oz/A and Leverage 360 4.0 oz/A; 24 Jul – Ranman 400SC 2.75 oz/A and Assail 30SG 2.5 oz/A; 6 Aug – Presidio 3.7 oz/A.
b. "Dyn-amic" surfactant included in spray solution at 0.25% (v/v) concentration.
c. "Induce" surfactant included in spray solution at 0.125% (v/v) concentration.
d. "Cohere" surfactant included in spray concentration at 0.03% (v/v) concentration.

Table S3. ANOVA test result of the DM infection imaging-derived severity rates at the panel level

	Df	Sum Sq	Mean Sq	F value	Pr(>F)
Treatment	5	3.937	0.7873	116.8	<2e-16
Residuals	42	0.283	0.0067		

Table S4. Tukey test result of the DM infection imaging-derived severity rates at the panel level

	diff	lwr	upr	p adj
2-1	0.001491776	-0.10867758	0.11166113	1.0000000
3-1	0.151969384	0.04180003	0.26213873	0.0075973
4-1	0.026711117	-0.08345823	0.13688047	0.9862273
5-1	0.061463800	-0.04870555	0.17163315	0.6677646
Ctl-1	0.804228140	0.69405879	0.91439749	0.0000000
3-2	0.150477608	0.04030826	0.26064696	0.0084136
4-2	0.025219342	-0.08495001	0.13538869	0.9893801
5-2	0.059972025	-0.05019733	0.17014138	0.6901407
Ctr-2	0.802736365	0.69256701	0.91290572	0.0000000
4-3	-0.125258266	-0.23542762	-0.01508892	0.0425555
5-3	-0.090505583	-0.20067493	0.01966377	0.2571673
Ctr-3	0.652258757	0.54208941	0.76242811	0.0000000
5-4	0.034752683	-0.07541667	0.14492203	0.9567519
Ctr-4	0.777517023	0.66734767	0.88768637	0.0000000
Ctr-5	0.742764340	0.63259499	0.85293369	0.0000000

Table S5. ANOVA test result of the DM infection imaging-derived severity rates at the spray unit level

	Df	Sum Sq	Mean Sq	F value	Pr(>F)
Treatment	5	2.3491	0.4698	78.99	1.32e-11
Residuals	18	0.1071	0.0059		

Table S6. Tukey test result of the imaging-derived DM infection severity rates at the spray unit level

	diff	lwr	upr	p adj
2-1	0.002812315	-0.15080021	0.15642484	0.9999999
3-1	0.168274840	0.01466232	0.32188736	0.0599399
4-1	0.029271048	-0.12434148	0.18288357	0.9937971
5-1	0.069093207	-0.08451932	0.22270573	0.7983945
Ctl-1	0.879366686	0.72575416	1.03297921	0.0000000
3-2	0.165462524	0.01185000	0.31907505	0.0662511
4-2	0.026458733	-0.12715379	0.18007126	0.9961199
5-2	0.066280891	-0.08733163	0.21989341	0.8238936
Ctl-2	0.876554370	0.72294185	1.03016689	0.0000000
4-3	-0.139003792	-0.29261631	0.01460873	0.1616644
5-3	-0.099181633	-0.25279416	0.05443089	0.4790470
Ctl-3	0.711091846	0.55747932	0.86470437	0.0000000
5-4	0.039822159	-0.11379036	0.19343468	0.9754477
Ctl-4	0.850095638	0.69648311	1.00370816	0.0000000
Ctl-5	0.810273479	0.65666096	0.96388600	0.0000000

Table S7. ANOVA test result of the DM infection human assessed severity rates

	Df	Sum Sq	Mean Sq	F value	Pr(>F)
Treatment	5	2.3380	0.4676	32.44	2.12e-08
Residuals	18	0.2594	0.014		

Table S8. Tukey test result of the DM infection human assessed severity rates

	diff	lwr	upr	p adj
2-1	0.02556818	-0.24421142	0.29534778	0.9996046
3-1	0.31321023	0.04343063	0.58298983	0.0176717
4-1	0.01278409	-0.25699551	0.28256369	0.9999870
5-1	0.15127841	-0.11850119	0.42105801	0.5002602
Ctl-1	0.88536932	0.61558972	1.15514892	0.0000001
3-2	0.28764205	0.01786245	0.55742164	0.0328067
4-2	-0.01278409	-0.28256369	0.25699551	0.9999870
5-2	0.12571023	-0.14406937	0.39548983	0.6798654
Ctl-2	0.85980114	0.59002154	1.12958073	0.0000001
4-3	-0.30042614	-0.57020573	-0.03064654	0.0241238
5-3	-0.16193182	-0.43171142	0.10784778	0.4290506
Ctl-3	0.57215909	0.30237949	0.84193869	0.0000330
5-4	0.13849432	-0.13128528	0.40827392	0.5898506
Ctl-4	0.87258523	0.60280563	1.14236483	0.0000001
Ctl-5	0.73409091	0.46431131	1.00387051	0.0000011

Table S9. ANOVA test result of the PM infection imaging-derived severity rates at the panel level

	Df	Sum Sq	Mean Sq	F value	Pr(>F)
Treatment	17	3.0370	0.17865	52.68	<2e-16
Residuals	110	0.3731	0.00339		

Table S10. ANOVA test result of the PM infection imaging-derived severity rates at the spray unit level

	Df	Sum Sq	Mean Sq	F value	Pr(>F)
Treatment	17	2.022	0.11893	63.65	<2e-16
Residuals	46	0.086	0.00187		

Table S11. ANOVA test result of the PM infection human assessed severity rates

	Df	Sum Sq	Mean Sq	F value	Pr(>F)
Treatment	17	2.4603	0.1447	45.21	<2e-16
Residuals	46	0.1472	0.0032		
